# Marital relationship, parenting practices, and social skills development in preschool children

**DOI:** 10.1186/s13034-016-0139-y

**Published:** 2017-01-07

**Authors:** Rikuya Hosokawa, Toshiki Katsura

**Affiliations:** Graduate School of Medicine, Kyoto University, Yoshida-Konoe-cho, Sakyo-ku, Kyoto, 606-8501 Japan

**Keywords:** Marital conflict, Marital relationship, Parenting practices, Preschool children, Social skills

## Abstract

**Background:**

This study examined the pathways by which destructive and constructive marital conflict leading to social skills development in preschool children, are mediated through negative and positive parenting practices.

**Methods:**

Mothers of 2931 Japanese children, aged 5–6 years, completed self-report questionnaires regarding their marital relationship (the Quality of co-parental communication scale) and parental practices (the Alabama parenting questionnaire). The children’s teachers evaluated their social skills using the Social skills scale.

**Results:**

Path analyses revealed significant direct paths from destructive marital conflict to negative parenting practices and lower scores on the self-control component of social skills. In addition, negative parenting practices mediated the relationship between destructive marital conflict and lower scores on cooperation, self-control, and assertion. Our analyses also revealed significant direct paths from constructive marital conflict to positive parenting practices, and higher scores on cooperation and assertion. Positive parenting practices mediated the relationship between constructive marital conflict and higher scores on self-control and assertion.

**Conclusions:**

These findings suggest that destructive and constructive marital conflict may directly and indirectly influence children’s social skills development through the mediation of parenting practices.

## Background

Research studies consistently suggest that marital conflict affects children’s social functioning and strongly predicts their adjustment, including internalizing disorders (e.g. anxiety, depression, and withdrawal), externalizing disorders (e.g. aggression, delinquency, and conduct disorders), and academic achievement [1–4].

Although marital conflict is associated with children’s development, few studies have explored how different types of marital conflict affect it. Marital conflict typically is classified into two categories: destructive and constructive. Destructive marital conflict involves behaviors, such as violence, nonverbal conflict, withdrawal during conflict, verbal aggression or hostility, aggression against objects, and threats to family integrity [2]. Exposure to destructive marital conflict precipitates children’s anger, worry, and sadness [5, 6]. Such conflict may put them at the risk for developing adjustment problems (internalizing and externalizing disorders), due to a lack of coping skills to deal with emotions, or the use of learned aggressive behavior for solving problems [1, 3, 7]. In contrast, constructive marital conflict involves successful conflict resolution, explanations of how conflict is resolved, and optimistic explanations of unresolved conflict [2]. Exposure to constructive conflict fosters positive emotional reactions, such as happiness, safety, and security [3, 5, 8]. Constructive marital conflict may help children develop problem solving, coping, and conflict resolution skills by teaching them problem solving and effective communication skills [4, 6, 9]. Studies consistently suggest that destructive conflict increases children’s risk for adjustment disorders, whereas constructive conflict increases positive adjustment. Despite the differential effects of destructive and constructive conflict on children’s development, studies examining the different types of conflict and their developmental implications are lacking. Accordingly, the aim of this study was to examine destructive and constructive marital conflict as predictors of child development.

Parenting practices may mediate the association between marital conflict and children’s development [2]. “Spillover” theories explaining the relationship between marital conflict and children’s functioning hypothesize that the negativity and positivity experienced in the inter-parental relationship transfer to the parent–child relationship [10, 11]. Parents engaged in destructive marital conflict often lack emotional availability and are less responsive to their children’s needs [12]. Limited research has been conducted on the impact of constructive marital conflict on positive parenting, as most studies have focused on destructive marital conflict. Few studies have examined whether constructive marital conflict fosters positive spillover, resulting in more positive parent–child interactions and child development. Studies have consistently found a negative association (negative spillover) between destructive marital conflict and children’s development, and negative parenting practices appear to mediate these relationships. Most studies have focused on negative rather than positive parenting practices with minimal consideration of constructive relations’ effects on marital conflict. Investigations of negative and positive parenting practices are needed to understand how marital conflict impacts child development.

Children’s school adjustment is particularly important and the inability to adjust socially due to poor social skills (e.g. cooperation, self-control, and assertion) is a factor in their maladjustment to school [13, 14]. Social skills development is essential to acquire social competence [15, 16]. Social skills deficits in early childhood are relatively stable over time, and may have negative consequences, including, internalizing disorders, externalizing disorders, and poor academic performance; these consequences may be precursors of more severe problems later in life [17–19]. The development of children’s social skills is significantly affected by their child-rearing environment [20]. Although marital conflict and parenting practices affect social competence [21, 22], few studies have investigated how these variables affect social skills in a comprehensive way. Thus, it is important to examine the associations among marital conflict, parenting practices, and social skills development in a comprehensive model.

### Current study

Few studies have examined destructive and constructive marital conflict to understand the differential effects of conflict styles on children’s social competence. Although many studies have found negative associations between destructive marital conflict and children’s development, and that negative parenting practices mediate these relationships, few have investigated the impact of constructive marital conflict on positive parenting and child development. In addition, research suggests that marital conflict and parenting practices affect social competence independently, yet few investigations have addressed how these variables affect social skills in a comprehensive model.

It is important to analyze independent associations while controlling for other variables in investigations of the relationships among these variables; however, studies have primarily examined the relationships between different types of marital conflict, parenting practices, and child outcomes without analyzing these other associations. Therefore, we aimed to clarify the roles of marital conflict (i.e. destructive and constructive marital conflict), parenting practices (i.e. negative and positive parenting practices), and children’s social skills development (i.e. cooperation, self-control, and assertion) by analyzing these relationships in a comprehensive model.

We hypothesized the following pathways: (1) an indirect pathway between destructive marital conflict and lower child’s social skills development mediated by negative parenting practices; (2) an indirect pathway between constructive marital conflict and higher child’s social skills development mediated by positive parenting practices; (3) a direct pathway between the destructive marital relationship and lower child’s social skills development, and (4) a direct pathway between constructive marital relationship and higher child’s social skills development, adjusting for parenting practices.

## Methods

### Participants

The present study is part of a regional investigation of the effects of child-rearing environment on children’s social development. In 2014, self-report questionnaires were given to parents of preschool children (*N* = 5024) aged 5–6 years in 52 kindergartens and 78 nursery schools in Nagoya city, which is a major urban area in Japan. The children’s mothers (*N* = 3273) completed the questionnaires. Mothers were required to be married and to reside with the child but were not required to be the child’s biological parent. Children from fatherless families and children diagnosed with a developmental problem were excluded from the study. Ethical approval for this study was obtained from Kyoto University’s Ethics Committee in Kyoto, Japan (E2322).

### Measures

#### Marital relationship (predictors)

The Quality of Co-parental Communication Scale (QCCS) is a 10-item self-report measure of each partner’s feelings or behaviors in the co-parenting relationship [23]. It has two subscales: Co-parental conflict (4 items reflecting conflict, hostility, tension, and disagreements) and Co-parental support (6 items reflecting accommodation, helpfulness, and resourcefulness). Items are rated on a 5-point scale ranging from 1 (never) to 5 (always). The conflict and support subscales assess parents’ perceptions of the co-parenting relationship, with higher conflict scores indicating higher co-parental conflictive communication (destructive conflict), and higher support scores indicating higher co-parental supportive communication (constructive conflict). The scales have adequate internal consistency and construct validity [23–25]: the internal consistency is 0.88 for conflict and 0.74 for support [23]. This study had internal consistencies of 0.79 for the Conflict scale and 0.87 for the Support scale. Each QCCS total score was converted to a z score.

#### Parenting practices (mediators)

The Alabama Parenting Questionnaire (APQ) is a 42-item self-report measure of parenting behavior with five subscales [26]: Poor monitoring/supervision, Inconsistent discipline, Corporal punishment, Positive parenting, and Involvement. Items are rated on a 5-point scale ranging from 1 (never) to 5 (always). The questionnaire has adequate internal consistency and construct validity. The internal consistency of the subscales ranges from 0.46 to 0.80 [26]. In this study, the subscales’ internal consistency ranged from 0.70 to 0.77. A Negative parenting composite score was calculated by converting the Poor monitoring/supervision, Inconsistent discipline, and Corporal punishment subscales to z scores and averaging the z scores; higher scores indicate higher negative parenting. A Positive parenting composite score was calculated using the same method for the Positive parenting and Involvement subscales, with higher scores indicating higher positive parenting.

#### Child’s social skills (criterion variables)

The Social Skills Scale (SSS) is a 24-item measure of children’s social competence in “cooperation,” “self-control,” and “assertion” [27–29], factors affecting social adaption in later life [14]. In this study, children’s teachers evaluated their social skills using this scale’s three subscales: Cooperation (8 items e.g. “Helps friends when asked”), Self-control (8 items e.g. “Postpones gratification when requested”), and Assertion (8 items e.g. “Expresses appropriate greetings to others”). These factors positively correlated with the child development scale [27], which is based on the social skills rating systems (SSRS) [14]. Items are rated on a 3-point scale ranging from 0 (not at all) to 2 (often), and total scores for cooperation, self-control, and assertion are obtained, with higher scores indicating higher social skills. The measure has adequate internal consistency and construct validity; the subscales’ internal consistency ranged from 0.91 to 0.93 [27]. In this study, the subscales’ internal consistency ranged from 0.83 to 0.93. Each SSS T-score was used in this study.

### Procedure

Participants were from 52 kindergartens and 78 nursery schools. The principal of each facility granted permission to contact parents. The researcher contacted the parents and distributed an information sheet and a questionnaire pack, which were identified by their child’s ID number. Parents completed the questionnaires and returned them in sealed envelopes via the participating facilities. Then, the child’s teacher checked the ID number, and evaluated the child’s social skills using the SSS.

### Data analyses

**Fig. 1:**
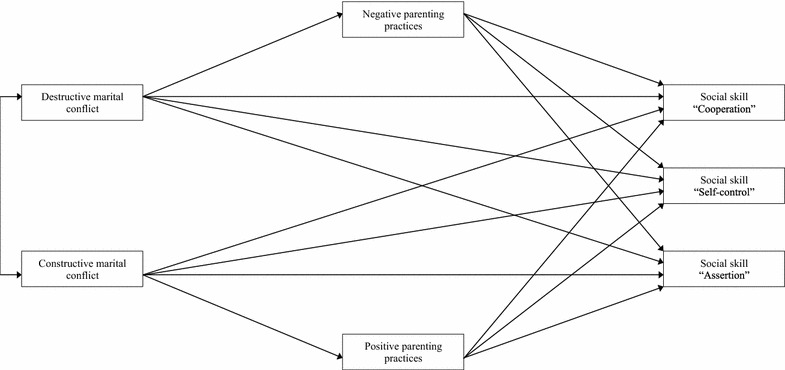
Hypothesized model. This model includes the hypothesized pathways between marital relationship (destructive and constructive marital conflict), parenting practices (negative and positive parenting practices), and children’s social skills (cooperation, self-control, and assertion)

Correlations were performed to measure associations between marital relationship (destructive and constructive marital conflict), parenting practices (negative and positive parenting practices), social skills (cooperation, self-control, assertion), and demographic characteristics (child’s sex and age). Path analyses were conducted to estimate direct and indirect paths between destructive marital conflict, constructive marital conflict, negative parenting practices, positive parenting practices, and children’s social skills. In the models, negative and positive marital relationships were specified as predictors of (a) negative and positive parenting practices, and (b) children’s social skills. Prior to estimating the full model (Fig. 1) to test the effects of a positive marital relationship and positive parenting practices, a reduced model that did not include these variables was estimated.

To assess fit, we used the comparative fit index (CFI) [30], the incremental fit index (IFI) [31], and the root mean square error of approximation (RMSEA) [32]. Good model fit is reflected in CFI and IFI values above 0.90 [30, 31] and RMSEA values of 0.08 or less [33]. All statistical analyses were conducted using SPSS version 22.0 and Amos version 23.0.

## Results

### Descriptive statistics

Of the 3273 mothers who completed questionnaires, data from 2931, which met the inclusion criteria, were analyzed. The children included 1476 males (50.4%) and 1455 females (49.6%). Their ages ranged from 5 to 6 years (M = 6.10, SD = 0.29), the mothers’ ages from 20 to 55 (M = 37.12, SD = 4.64), and the fathers’ ages ranged from 22 to 67 (M = 39.12, SD = 5.63). Children attending kindergartens totaled 1315; 1616 children attended nursery schools.

Descriptive statistics for all study variables are presented in Table 1 and the correlation results for the composite measures are presented in Table 2. All correlations between the composites were statistically significant. Significant correlations were found between certain demographic variables and outcome variables reflecting children’s social skills, namely, cooperation, self-control, and assertion. Children’s sex and age were significantly related to social skills (cooperation, self-control, and assertion). Therefore, these variables were entered in the predictive models as controls.

**Table 1:** Descriptive statistics for the study variables (*N* = 2931)

Description	Range (min–max)	M	SD	α
Quality of co-parental communication scale (QCCS)				
Co-parental conflict	4–20	10.04	3.02	0.79
Co-parental support	6–30	25.00	4.20	0.87
Alabama parenting questionnaire (APQ)				
Poor monitoring/supervision	10–50	12.88	4.12	0.70
Inconsistent discipline	6–30	14.61	3.71	0.73
Corporal punishment	3–15	7.10	2.14	0.72
Positive parenting	6–30	22.33	3.47	0.77
Involvement	10–50	37.82	5.12	0.76
Social skills scale (SSS)				
Cooperation	0–16	10.98	4.19	0.93
Self-control	0–16	14.18	2.54	0.89
Assertion	0–16	14.09	2.32	0.83

**Table 2:** Correlations between the types of marital relationships, parenting practices, children’s social skills, and demographic characteristics

Variable	1	2	3	4	5	6	7	8	9
Marital relationship									
1. Destructive marital conflict	–								
2. Constructive marital conflict	−0.61***	–							
Parenting practices									
3. Negative parenting practices	0.27***	−0.19***	–						
4. Positive parenting practices	−0.17***	0.27***	−0.28***	–					
Child social skills									
5. Cooperation	−0.06***	0.09***	−0.10***	0.04*	–				
6. Self-control	−0.10***	0.08***	−0.16***	0.08***	0.39***	–			
7. Assertion	−0.07***	0.08***	−0.11***	0.09***	0.56***	0.42***	–		
Demographic characteristics									
8. Sex	−0.01	0.00	−0.08**	0.00	0.18***	0.25***	0.13***	–	
9. Age	0.05*	−0.03	0.04	−0.05*	0.12***	0.04*	0.07***	−0.02	–

[Marital relationship] Destructive marital conflict: QCCS Co-parental conflict. Constructive marital conflict: QCCS Co-parental support. [Parenting practices] Negative parenting practices: APQ Poor monitoring/supervision, Inconsistent discipline, Corporal punishment. Positive parenting practices: APQ Positive parenting, Involvement. [Demographic characteristics] Sex (0 = male, 1 = female)

* *p* < 0.05; ** *p* < 0.01; *** *p* < 0.001

### Hypothesized paths

Model 1 did not include constructive marital conflict and positive parenting practices (Table 3). Destructive marital conflict significantly predicted of negative parenting practices (β = 0.26, *p* < 0.001), children’s cooperation (β = −0.04, *p* < 0.05), self-control (β = −0.07, *p* < 0.001), and assertion (β = −0.05, *p* < 0.05).

**Table 3:** Unstandardized and standardized coefficients for path analyses

Model/construct			B	SE	β	*p*
Model 1						
Destructive marital conflict	→	Negative parenting practices	0.17	0.01	0.26	***
Destructive marital conflict	→	Social skill “Cooperation”	−0.44	0.19	−0.04	*
Destructive marital conflict	→	Social skill “Self-control”	−0.73	0.18	−0.07	***
Destructive marital conflict	→	Social skill “Assertion”	−0.47	0.19	−0.05	*
Negative parenting practices	→	Social skill “Cooperation”	−1.13	0.29	−0.07	***
Negative parenting practices	→	Social skill “Self-control”	−1.78	0.28	−0.12	***
Negative parenting practices	→	Social skill “Assertion”	−1.27	0.29	−0.08	***
Model 2						
Destructive marital conflict	→	Negative parenting practices	0.16	0.02	0.24	***
Destructive marital conflict	→	Positive parenting practices	−0.01	0.02	−0.02	
Destructive marital conflict	→	Social skill “Cooperation”	0.03	0.23	0.00	
Destructive marital conflict	→	Social skill “Self-control”	−0.60	0.23	−0.06	**
Destructive marital conflict	→	Social skill “Assertion”	−0.08	0.23	−0.01	
Constructive marital conflict	→	Negative parenting practices	−0.03	0.02	−0.04	
Constructive marital conflict	→	Positive parenting practices	0.21	0.02	0.25	***
Constructive marital conflict	→	Social skill “Cooperation”	0.77	0.23	0.08	***
Constructive marital conflict	→	Social skill “Self-control”	0.15	0.23	0.02	
Constructive marital conflict	→	Social skill “Assertion”	0.55	0.23	0.05	*
Negative parenting practices	→	Social skill “Cooperation”	−1.07	0.29	−0.07	***
Negative parenting practices	→	Social skill “Self-control”	−1.65	0.29	−0.11	***
Negative parenting practices	→	Social skill “Assertion”	−1.05	0.30	−0.07	***
Positive parenting practices	→	Social skill “Cooperation”	0.12	0.23	0.01	
Positive parenting practices	→	Social skill “Self-control”	0.42	0.23	0.04	*
Positive parenting practices	→	Social skill “Assertion”	0.67	0.24	0.06	**

* *p* < 0.05; ** *p* < 0.01; *** *p* < 0.001

Model 2 included constructive marital conflict and positive parenting practices as additional predictors of children’s social skills (Table 3). Destructive marital conflict significantly predicted negative parenting practices (β = 0.24, *p* < 0.001) and children’s self-control (β = −0.06, *p* < 0.01). Negative parenting practices significantly predicted children’s cooperation (β = −0.07, *p* < 0.001), self-control (β = −0.11, *p* < 0.001), and assertion (β = −0.07, *p* < 0.001). In contrast, constructive marital conflict significantly predicted positive parenting practices (β = 0.25, *p* < 0.001), children’s cooperation (β = 0.08, *p* < 0.001), and assertion (β = 0.05, *p* < 0.05). Moreover, positive parenting practices significantly predicted the children’s self-control (β = 0.04, *p* < 0.05) and assertion (β = 0.06, *p* < 0.01).

**Fig. 2:**
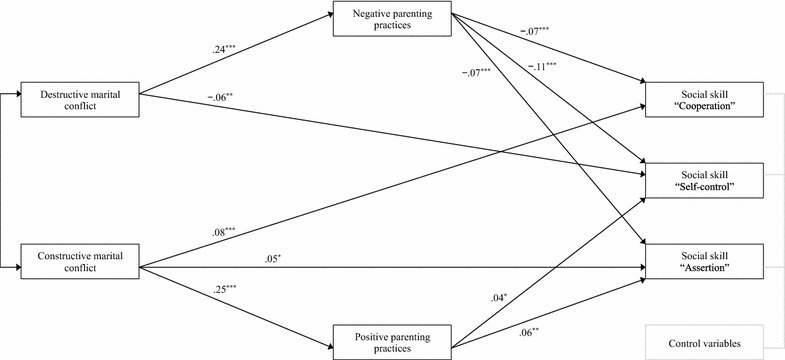
Statistically significant paths. This model includes the paths that were statistically significant in the hypothesized model. Path analyses controlled for child’s sex and age. Model fit statistics: χ^2^ (9) = 28.80; CFI = 1.00; IFI = 1.00; RMSEA = 0.03. *p < 0.05; **p < 0.01; ***p < 0.001

Both destructive and constructive marital types of conflict were significantly associated with children’s social development through negative and positive parenting practices. These models had a good fit with the data (model 1 fit statistics: χ^2^ (5) = 25.07, *p* < 0.001; CFI = 0.99; IFI = 0.99; RMSEA = 0.04, model 2 fit statistics: χ^2^ (9) = 28.80, *p* < 0.001; CFI = 1.00; IFI = 1.00; RMSEA = 0.03). Although both models met the criteria for acceptable fit, model 2’s fit statistics were better than those of model 1. Therefore, model 2 displays the final model and the standardized path coefficients (Fig. 2).

## Discussion

This study examined the associations and mediation effects between marital relationship, parenting practices, and social skills in preschool children. Destructive and constructive marital conflict significantly predicted the development of social skills indirectly through parenting practices. Destructive and constructive marital conflict significantly predicted the development of social skills directly, adjusting for parenting practices. These findings support the model suggested by the researcher.

### Indirect path between variables

#### Association between marital conflict and parenting practices

Parents’ destructive conflict was associated with lower cooperation, self-control, and assertion indirectly, through negative parenting practices. However, exposure to constructive conflict was associated with higher self-control and assertion indirectly, through positive parenting practices. This result is consistent with previous research identifying associations between destructive marital conflict and negative parenting practices [34–36]. This result supports the positive spillover effect, as constructive marital conflict was related to higher levels of positive parenting. Similar to negative spillover effects and consistent with family systems theory [12], positive emotions from inter-parental relationships may transfer to parent–child relationships [10, 11].

#### Association between parenting practices and child’s social skills

The association between parenting practices and children’s social skills (cooperation, self-control, and assertion) is consistent with previous research findings [37]. Negative parenting, such as talking less or more harshly to children, is likely to rob children of opportunities to acquire social skills through interactions with others. However, positive parenting, such as talking to children warmly or supportively, may provide opportunities to gain social skills through interactions with others. As children develop, social competence increasingly depends on the ability to use language to communicate needs and desires and to negotiate tasks [38]. Negative or positive parenting practices may influence children’s opportunities to communicate and understand their own and others’ intentions and feelings, thus, limiting or enhancing their ability to negotiate conflicts to reach prosocial solutions [39]. Over time, parenting practices may influence social interactions and opportunities to gain social competence, such as emotional expression and communication skills. In addition, social skills development, i.e. emotional expression and communication skills, may influence children’s ability to control their emotions [40]. Therefore, destructive and constructive marital conflict are likely to influence negative and positive parenting practices directly, and the development of social skills.

### Direct path between marital conflict and social skills

Parents’ destructive conflict was directly associated with lower self-control, whereas constructive conflict was directly associated with higher cooperation and assertion. These results are consistent with previous studies indicating that exposure to marital conflict is associated with different responses in children, depending on how parents negotiate their differences [2, 3].

Modeling has been identified as an important direct mechanism by which parental relationships affect children’s development. Consistent with the modeling mechanism proposed by the spillover hypothesis, children directly learn behaviors exhibited by parents during marital disputes. Children of parents who resolve problems through aggressive behavior are more likely to learn aggression as an acceptable way of dealing with disagreements and to act aggressively when interacting with peers [4]. However, children of parents who resolve problems through cooperation are more likely to learn the negotiation skills they observe between parents and to communicate more effectively with peers [6]. In addition, an important direct mechanism of destructive parental relationships on children’s development is the cumulative effect of exposure to chronic stress. Children from families with destructive marital conflict are likely to be exposed to physical and psychosocial stress from domestic disturbances, such as disputes and violence. As exposure to stressors accumulates, physiological response systems designed to handle relatively infrequent, acute demands are overwhelmed. Chronic cumulative stressors disrupt self-regulatory processes that help children cope with external demands [41, 42]. Therefore, destructive and constructive marital conflict is likely to influence social skills development directly.

### Limitations and future directions

Our findings should be interpreted in light of several limitations. First, it used a cross-sectional design, whereas longitudinal designs are needed to examine the effects of marital relationships on the later development of preschool children. Second, only mothers completed the questionnaires; however, fathers’ reports are needed to evaluate more accurately, both parents’ involvement in their children’s rearing. Finally, the sample was recruited from a limited area of an urban metropolis; thus, the reproducibility of the results should be confirmed using data from other settings. Future studies would benefit from longitudinal designs and samples with greater demographic and clinical diversity.

## Conclusion

This study found significant direct paths from destructive marital conflict to negative parenting practices and social skills, and significant direct paths from constructive marital conflict to positive parenting practices and social skills. These findings offer preliminary evidence of the need to explore negative and positive aspects of family relationships. They advance our understanding of marital conflict and parenting using a family systems explanation for children’s development. A lack of social skills in early childhood places children at risk for social maladjustment [13, 18, 19]. Therefore, a simultaneous focus on marital conflict and parenting in negative and positive domains may be an effective strategy for developing children’s social adjustment.

## Abbreviations

QCCS: the Quality of Co-parental Communication Scale
APQ: the Alabama Parenting Questionnaire
SSS: the Social Skills Scale
